# The impacts of expertise, conflict, and scientific literacy on trust and belief in scientific disagreements

**DOI:** 10.1038/s41598-025-96333-8

**Published:** 2025-04-07

**Authors:** Natasha van Antwerpen, Estelle B. Green, Daniel Sturman, Rachel A. Searston

**Affiliations:** School of Psychology, The University of Adelaide, North Terrace, Adelaide, SA 5005 Australia

**Keywords:** Trust in science, Trust in experts, Science communication, Scientific disagreement, Scientific literacy, Psychology, Human behaviour

## Abstract

Media portrayals of scientific disagreements can blur distinctions between experts and non-experts, or include disagreements from vested individuals, potentially undermining trust in science and belief in scientific claims. We investigated how disagreeing sources’ expertise and conflicting interests impact trust in scientific experts and belief in their claims, and whether scientific literacy moderates these effects. Across three, 2 × 2 factorial experiments with a student (*N* = 105) online (*N* = 110), and general Australian sample (*N* = 105), participants read articles describing a scientific claim followed by a disagreeing source whose subject-matter expertise (present/absent) and vested interest (present/absent) were manipulated. Participants in all samples judged the original scientific expert as more trustworthy and their claims more believable when the disagreeing source lacked relevant subject-matter expertise. Among student participants, conflicts of interest also impacted belief in scientific claims (but not trust in the scientist), and scientific literacy enhanced sensitivity to expertise and conflict, however, the other samples were largely insensitive to vested interests, and scientific literacy had varied effects in these samples. Our results show disagreement in the news, even from questionable sources, can sway evaluations of scientific claims and scientists, and highlight the value of literacy-based interventions in science communication.

## Introduction

Scientific claims can hold significant implications for public safety and well-being. Approximately 97% of climate scientists agree on human-caused climate change^1^, and consensus among epidemiologists supported the efficacy of social distancing, mask-wearing, and vaccination in reducing COVID-19 transmission rates^2^. However, the public often access such claims through social and news media^3,4^, which can amplify voices contradicting scientific consensus without giving indications to subject-matter expertise, or vested interests^5,6^. These dissenting perspectives can increase perceived uncertainty, diminish trust, and reduce belief in scientists and their assertions^7–9^, leading to negative downstream effects, including less support and adherence to protective behaviours during COVID-19^10,11^.

Existing work demonstrates characteristics of individuals *making* science-based claims, including expertise and conflicting interests, impact evaluations of claimants and their statements^12–16^. However, the influence of *disagreeing* individuals’ characteristics is less understood. Disagreeing views are readily available in modern media landscapes and, alongside misinformation, make distinguishing true scientific claims challenging^17^. Journalistic devices like ‘false balance’ give equal weight to opposing claims without reference to expertise, evidence, or vested interest^18^. These factors shape perceptions of scientific facts^19^, scientific consensus^20^, and contribute to information overload^21^. While education and efforts to increase scientific literacy can elevate public comprehension of science news, a full and comprehensive understanding of all scientific knowledge is impractical. Consequently, (dis)trust becomes a crucial heuristic in evaluating scientific claims^22^; or alternative explanations like conspiracy theories and misinformation^23^.

Improving public understanding of science, therefore requires identifying factors influencing trust in experts and belief in scientific claims. Reporting characteristics of those *disagreeing* with scientific claims could help assess the credibility of opposing arguments on science-based issues. We therefore examine how *disagreeing* individuals’ characteristics influence trust in *claiming* scientists and belief in their claims. Since characteristics of the trustor (individuals evaluating the claim) also affect trust and belief^24^, we investigate scientific literacy as a moderator.

### Trust in experts

Though closely related^4^, an individual might trust a scientific expert without trusting science generally, or vice versa. We focus on trust in scientific experts rather than trust in science itself. By scientific expert we refer to someone who has developed skills, abilities, and/or knowledge in a specialised scientific domain, including certain modes of knowledge production^25^.

Though challenging to define and measure^26^, trust is considered an attitude or belief where the trustor accepts a degree of vulnerability by being willing to believe and act on the actions, beliefs, or decisions of the trustee^24^. Theories of trust can therefore focus on trustor characteristics, trustee characteristics, or both. Similarly, theories often consider belief and behavioural components of trust, with beliefs (how trustworthy someone/something is) influencing behavioural outcomes (willingness to act).

We draw on Mayer et al.’s (1995) ABI model of trust, which conceptualises trust as influenced by perceptions of the trustee’s Ability, Benevolence, and Integrity^24^. Consistent with the ABI, a review found competence, integrity, benevolence, and openness are four distinct dimensions predicting trust in science with high explanatory power^27^. Accordingly, we manipulate two of these dimensions with demonstrated explanatory power and theoretical relevance for influencing trust and belief following scientific disagreements: Ability/competence, conceptualised as expertise consistent with previous research^28^; and integrity, conceptualised as conflict of interest (conflict), consistent with Merton’s scientific norm of disinterestedness, or the lack of self-interested motivations including personal or financial gain potentially incentivising tmanipulation or biasing of results^3,13^. These dimensions are more readily amenable to manipulation within the study context (news articles on scientific claims), while benevolence is more subjective, difficult to convey, and often less apparent in reporting. In scientific disagreements, expertise and conflict are also important factors influencing the motives and credibility of disagreeing individuals.

Previous experimental research has manipulated the *claimant’s* expertise, including whether they are a scientist or politician^29^, a domain or non-domain specific expert^16^, an expert or lay person^7^, or whether they provide a cue to expertise^30^, finding that (domain-specific) experts are perceived more trustworthy, and their claims more credible. However, the impact of *disagreeing* individuals’ characteristics on claimant trustworthiness remains unexamined. We therefore manipulate the *domain-specific* expertise of the *disagreeing* source, rather than the original claimant. While manipulating domain-specific expertise may result in smaller effects compared with general expertise, it reflects a common trend in news, where experts are requested to comment on topics and claims outside their domain-specific expertise^31^.

Like expertise, previous work has manipulated vested interests, often by having the claimant be a scientist or lobbyist, to examine effects on trustworthiness and credibility of claims. Findings have been mixed; some studies found no effect on trustworthiness^14^, or belief^13^. However, most work has found claimants are rated less trustworthy and their information less credible when described as lobbyists or from invested institutions than scientists or from university/research institutes^13,15^. Poll and survey data also suggests US and UK residents trust scientists more than other sources, such as news and politicians, particularly when employed by institutions and charities rather than private industry^32^.

While these findings indicate audiences are usually sensitive to expertise and conflict when scientists make claims, previous studies either do not investigate disagreements, or manipulate *claimant* characteristics only. In many news contexts, however, audiences may be equally influenced by characteristics of the disagreeing source and claimant, and disagreements may come from people with conflicts, or lacking expertise. Accordingly, we examine how characteristics of the *disagreeing* individual, including their subject-matter expertise and conflict, impact trust in the claiming scientist *and* belief in the claim. While we used a 2 × 2 factorial design, as the domains are usually distinct, we did not predict an interaction between conflict and expertise.

### Scientific literacy

Trust is also impacted by trustor characteristics, with scientific literacy likely to influence evaluations of scientists and their claims. Scientific literacy describes individuals’ ability to understand and apply scientific concepts, and to develop scientific skills, including critically analysing science claims and factors influencing their legitimacy, such as domain-specific expertise and vested interests^28,33^. Scientific literacy is often measured through knowledge of scientific facts and claims, though the concept includes understanding and communicating about science^34^.

In surveys reviewing scientific forecasts, participants reporting lower education, cognitive ability, and self-reported domain knowledge were more likely to attribute scientific disagreements to expert incompetence^35,36^. Comparatively, participants with higher education and cognitive ability were more likely to attribute disagreement to complexity, randomness, and expert bias, while those higher in self-reported domain knowledge were more likely to attribute disagreement to expert bias only. Individuals higher in scientific literacy may therefore be more sensitive to source information, such as domain-expertise or conflict, when evaluating expert trustworthiness and scientific claims. However, this has not been tested with experimental manipulation of source characteristics.

### Current study

We conducted an experiment and two replications investigating whether the characteristics of *disagreeing* individuals (expertise and conflict) influence people’s judgements of trust and belief in scientific claims. Building on past work^35,36^, we tested whether scientific literacy moderated the effects of conflict and *subject-matter* expertise on these evaluations, with the subtle manipulation of expertise intended to reflect how experts are often asked to comment on topics outside their domain in science news. We measured trust in the *claiming* scientist, and belief in their claim.

Past literature and theory suggest trust and belief in a claim will be *less* impacted by opinions from non-experts and those with vested interests^12–16^. Individuals with greater scientific literacy may be more sensitive to these characteristics^35,36^. We therefore predicted:

#### H1A

The *claiming* scientist will be rated *less* trustworthy when the *disagreeing* individual has no conflict, than when they have a conflicting interest.

#### H1B

Scientific literacy will moderate conflict’s impact such that, as people’s scientific literacy increases, conflict will have a greater impact on the perceived trustworthiness of the *claiming* scientist.

#### H2A

The *claiming* scientist will be rated *less* trustworthy when the *disagreeing* individual is a domain-expert, than when they are not a domain-expert.

#### H2B

Scientific literacy will moderate expertise’s impact such that, as people’s scientific literacy increases, the *disagreeing* individual’s expertise will have a greater impact on the perceived trustworthiness of the *claiming* scientist.

#### H3A

Belief in scientific claims will be lower when the *disagreeing* individual has no conflict, than when they have conflicting interests.

#### H3B

Scientific literacy will moderate conflict’s impact such that, as people’s scientific literacy increases, conflict will have a greater impact on people’s belief in the claim.

#### H4A

Belief in scientific claims will be lower when the *disagreeing* individual is a domain-specific expert, than when they are not a domain expert.

#### H4B

Scientific literacy will moderate expertise’s impact such that, as people’s scientific literacy increases, the *disagreeing* individual’s expertise will have a greater impact on people’s belief in the claim.

#### RQs

Is there an interaction between expertise and conflict, such that when the *disagreeing* individual lacks expertise and has a conflict this will impact trust in the *claiming* scientist (RQ1) and belief in the claim (RQ2) more than either factor in isolation?

## Experiment one methods

### Sample

A convenience sample testing the study idea was recruited from a student research pool in exchange for course credit. Participants comprised 105 (*n* = 20 male; *n* = 85 female) first-year Psychology students, with a mean age of 19 years (*SD* = 1.33). In an a priori power analysis for the predicted interaction effects (assuming a small effect size of *η²* = 0.10 and α = 0.05) conducted using G*Power 3.1, results indicated that a sample of 88 participants would be required to achieve a power of 0.95. We therefore aimed to recruit as many participants as possible between 8 August 2022 and 25 August 2022, with 88 participants as a minimum.

### Design

**Fig. 1:**
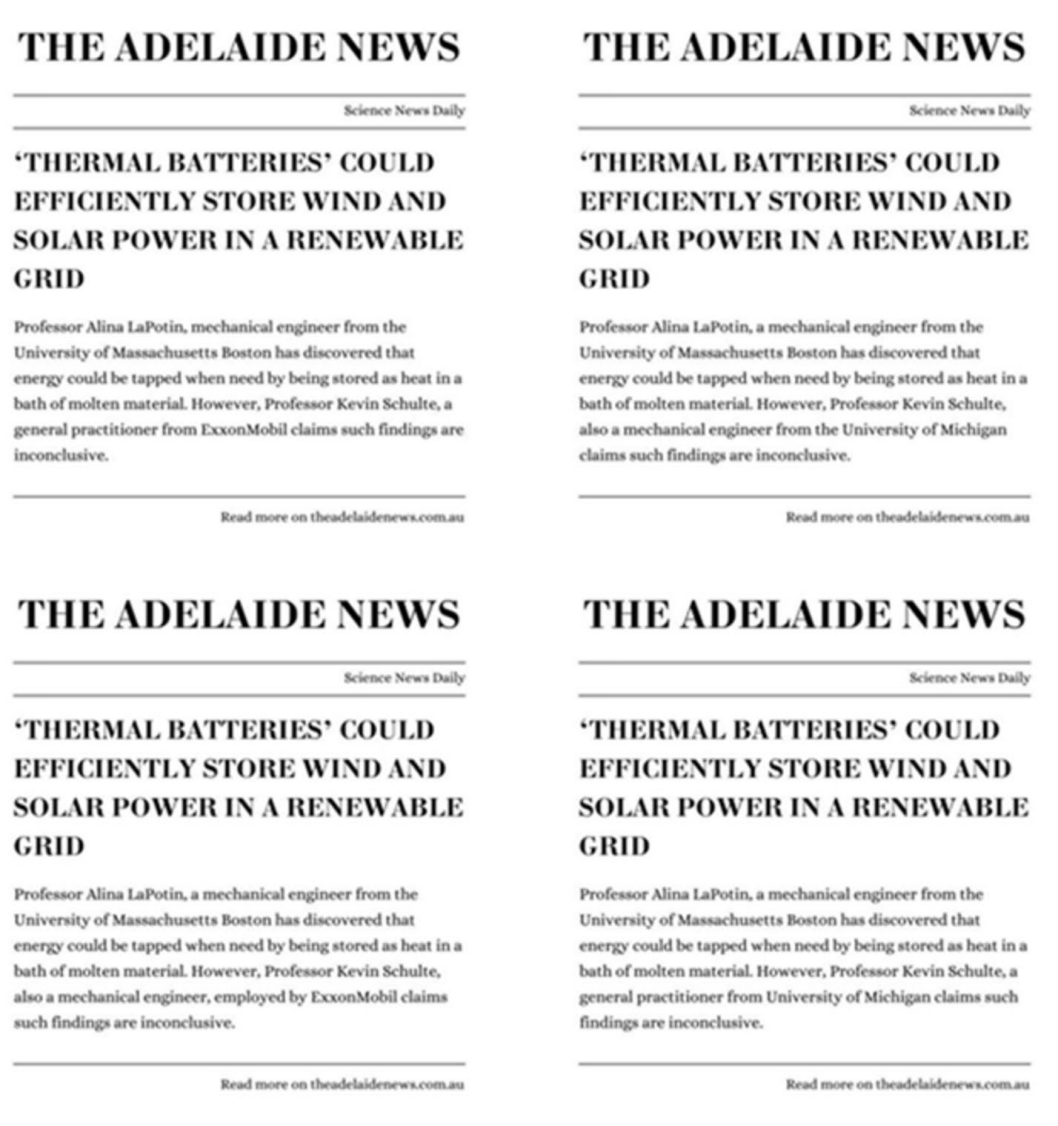
An example of an article in each condition. Non-expert, conflict of interest (lower left); expert, no conflict of interest (upper right); expert, conflict of interest (lower left); non-expert, no conflict of interest (lower right).

The study used a within-participants 2 (Conflict: vested interest present or absent for the individual expressing disagreement) × 2 (Expertise: subject matter expertise presence or absent for individual expressing disagreement) repeated-measures factorial experiment. Each participant read and responded to 16 short news articles (e.g., Fig. 1), where a scientific expert is quoted making a science-based claim within their area of expertise, and another individual is quoted disagreeing with the claim. In eight articles, the disagreeing individual has a vested interest (e.g., “Professor Dongmei Wang, also a biomedical scientist from Shenzhen Caibo Biological Technology, a producer of weight loss pills”), in the other eight no vested interest is mentioned (e.g., “Professor Dongmei Wang, also a biomedical scientist from University of Chinese Academy of Sciences”). Likewise in eight articles, the disagreeing individual has relevant domain expertise (e.g., “Professor Robert Sparks, an evolutionary ecologist”; Fig. 1) and in the other eight they have expertise in a different domain (e.g., “Professor Robert Sparks, a microbiologist”; Fig. 1). In each case, we kept the title of the claiming and disagreeing individual identical. While participants may consider disagreeing individuals with similar titles (e.g., Professor) to have more informed opinions than a general population, we wanted to ensure the manipulation was of the disagreeing individuals’ *domain-specific* expertise and conflict of interest, without also manipulating their title or seniority. This method of manipulating the traits of the disagreeing individual also aligned with the broader aim of the study, as people in positions of authority or expertise are often asked to comment on issues beyond the scope of their expertise, or which they may have a vested interest in.

We counterbalanced articles using a Latin Square approach, such that each article was presented with each manipulation equally, but individual participants only saw each claim once. This design enabled us to aggregate each participant’s trust and belief ratings over eight distinct claims, affording greater generalisability and power to detect the predicted effects. The presentation of stimuli varied within each block, meaning participants saw variations of the manipulation (expert/non-expert; conflict/no-conflict) in a random order. Such repeated-measures designs are a gold standard in cognitive science because they minimise the impact of random error variance by allowing participants to serve as their own control^37^. Taking multiple, ‘repeated’ measurements of trust and belief from each participant within each condition offers more power and a more robust assessment of cognitive states. The use of multiple short text-based scenarios as trails in such designs are common in studies of belief and trust, including for news and social media communication^38,39^. The randomisation and counterbalancing of stimuli, and variation in claim topics, made it unlikely participants understood the study intention.

### Stimuli

The stimuli consisted of 16 fictional news articles with genuine science-based headlines from article titles published in *Nature* or *Science*. We selected claims that were clear, politically neutral, accessible to a lay audience, and collectively covering a broad range of topics and scientific disciplines. We presented neutral rather than controversial claims, as most scientific claims in news media are relatively neutral^40^, and belief and trust in science claims could be influenced by prior beliefs or positions on controversial issues^41^.

Articles were shortened to approximately 50 words each, consisting of the headline, a statement of claim by a reporting scientist, and the claimant’s details. Claimant details were taken directly from the original *Science* and *Nature* articles and remained constant across all four experimental conditions. A sentence was added to each article stating disagreement with the original claim. This sentence was always expressed as: “However, **expert name**, an **expert occupation** from **affiliation**, claims such findings are debatable”. From here we created four versions of each article, varying this text (bolded above for clarity, not bolded in the articles) in-line with the four experimental conditions (Fig. 1).

The disagreeing individual in each article was given an affiliation that presented a vested interest or not (conflict manipulation) and occupation that demonstrated their domain expertise or lack thereof (Expertise manipulation). Conflict was manipulated such that disagreeing sources had a conflicting interest that was either monetary in nature (i.e., the claim negatively impacted their business or funding) or personal in nature (i.e., they had a vested interest in an opposing scientific theory), or they had no such interest. We varied conflict of interest between monetary or personal conflicts, as some claims had no clear monetary incentive, and having an opposing theory is a common, though more subtle, vested interest within scientific debates. While our manipulation in general was more subtle, this was in line with the aims of our study, which considered how people respond to cues to domain-specific expertise and conflicts of interest in news containing scientific disagreements. The seniority of the individuals involved was kept constant (e.g., both professors), as we were interested in manipulating domain-specific expertise and conflict of interest only. Final versions of each article were visually constructed to appear like newspaper articles in a fictitious newspaper (“The Adelaide News”).

An example of an article in each condition. Non-expert, conflict of interest (upper left); expert, no conflict of interest (upper right); expert, conflict of interest (lower left); non-expert, no conflict of interest (lower right).

## Measures

### Scientific literacy

Scientific literacy was measured using the eight-item scale from Kahan et al.^41^, adapted from the conventional scientific literacy questionnaire^34^. Six items contain common scientific knowledge (e.g., “Antibiotics kill viruses as well as bacteria”), with “True” or “False” response options. Two items contain an alternative answer (Earth/Sun) or (one day, one month, one year), with presentation of the second item contingent on a correct answer for the first. The closed question format is better suited to scientific questions asked in a survey or experimental format.

### Trust in reporting scientists

After each article, participants rated the trustworthiness of the reporting scientist: e.g., “How trustworthy is *Professor Dongmei Wang*?” from 0 = *‘not trustworthy at all’* to 10 = *‘completely trustworthy’*.

### Belief science claims

After each article, participants rated their belief in the original scientific claim: e.g., *“To what extent do you believe that moonlit nights lure the northern black swift to altitudes of more than 4*,*000 metres*,* which is higher than they soar on nights when the moon is new?*” from 0 = *‘don’t believe at all’* to 10 = *‘completely believe’*.

### Procedure

Participants completed the study online through Qualtrics. Participants were instructed that they would read and respond to a series of science articles, and to spend as much time as they wished reading each article before responding to it. They were then presented with the 16 articles in random order. After each article, participants rated the perceived trustworthiness of the *claiming* scientist, and belief in the claim. Participants also completed a multiple-choice question after each article to ensure they were attentive to the article and manipulation. The question focus varied across the claim, claimant, and disagreeing individual’s characteristics, reducing the possibility of cueing participants to the manipulation. All questions were scored as correct (1) or incorrect (0) with scores combined to form a total from 0 to 16. After reading and responding to all 16 articles, participants completed the scientific literacy scale and reported demographics (age, gender, education).

### Ethics

Ethics approval (number H-2022-58) was granted by the Human Research Ethics Subcommittee for low-risk research studies. Informed and voluntary consent was provided by all participants. Participation was anonymous, and participants were free to withdraw from the study at any stage. All methods were performed in accordance with the relevant guidelines and regulations.

### Data analysis

All data analyses were conducted in R. We examined effects using linear mixed effects models with the afex package in R, and tested significance with the car Anova function. We ran the analyses controlling for the claim and individual variance, with scientific literacy and the two manipulated variables entered as a three-way interaction e.g., Trust ~ Expertise*Conflict*Scientific Literacy + (1|Participant) + (1|Claim). The de-identified data, R script, outputs, and materials, is also openly available on OSF: https://osf.io/9kbe4/.

## Results

Multiple-choice scores suggested moderate retention of article details (*M* = 10.49, *SD* = 3.14).

### Trustworthiness of claiming scientist

Consistent with H1A, we found a significant small effect of conflict on trust (*β* = 1.68, *SE* = 0.41, *d* = 0.15, *p* < .001). When the *disagreeing* individual had a conflict, trust in the *claiming* scientist was higher (*M* = 6.32, *SD* = 1.88) than when they had no conflict (*M* = 6.05, *SD* = 1.80).

Consistent with H2A, there was a significant moderate effect of expertise on trust (*β* = -0.05, *SE* = 0.41, *d* = 0.39, *p* < .001). When the *disagreeing* individuals had expertise unrelated to the scientific claim, the *claiming* scientist was rated more trustworthy (*M* = 6.54, *SD* = 1.51), than when the *disagreeing* individual had domain-specific expertise (*M* = 5.83, *SD* = 1.49).

**Fig. 2:**
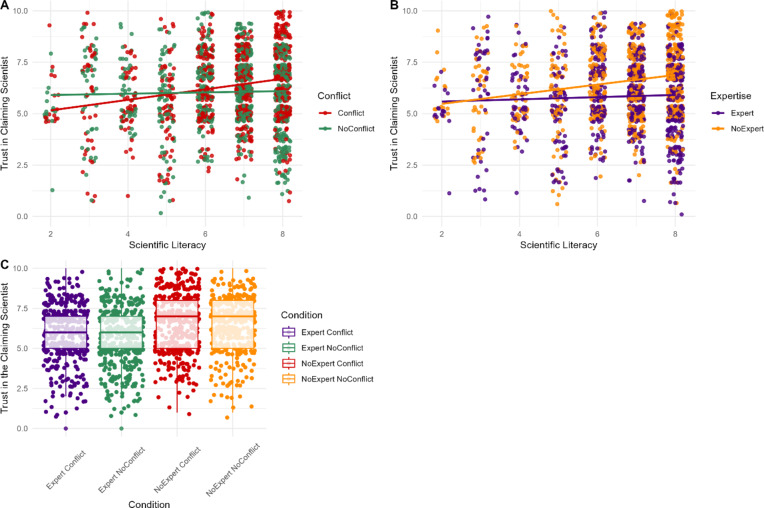
Participants’ ratings of trust in the reporting scientist based on their level of scientific literacy and the presence of a vested interest (**A**) or expertise (**B**), and by conflict and expertise (**C**) for the *disagreeing* individual. *Note*. For panel (**A**) the red points indicate individual participants mean trust scores when the individual disagreeing with the scientist’s claim had a conflict of interest, whereas the green points depict their mean trust scores when they did not have a conflict of interest. Similarly, for panel (**B**) orange points represent participant responses when the individual disagreeing with the scientist’s claim lacked relevant expertise, whereas purple points represent participant responses when they possessed relevant expertise.

Consistent with H1B and H2B, the interactions between expertise and scientific literacy (*β* = 0.11, *SE* = 0.06, *p* < .001), and conflict and scientific literacy (*β* = -0.30, *SE* = 0.06, *p* < .001), were significant. The effect of the *disagreeing* individuals’ vested interest or domain-relevant expertise was moderated by scientific literacy such that only those with greater scientific literacy were sensitive to conflicts or domain-specific expertise (Fig. 2). The interaction between conflict and expertise was non-significant when predicting trust (RQ1; *p* = .088).

### Belief in claim

H3A was supported, as conflict had a significant small main effect on belief (*β* = 1.61, *SE* = 0.50, *d* = 0.21, *p* < .001). When the *disagreeing* individual had no vested interest, belief in the claim was lower (*M* = 5.67, *SD* = 1.57), than when they had a vested interest (*M* = 6.06, *SD* = 1.55).

H4A was supported, with a significant small-moderate effect of expertise on belief (*β* = -0.86, *SE* = 0.49, *d* = 0.32, *p* < .001). When the *disagreeing* individual possessed domain-specific expertise participants’ belief ratings were lower (*M* = 5.54, *SD* = 1.50) than when they lacked domain-specific expertise (*M* = 6.19, *SD* = 1.59).

**Fig. 3:**
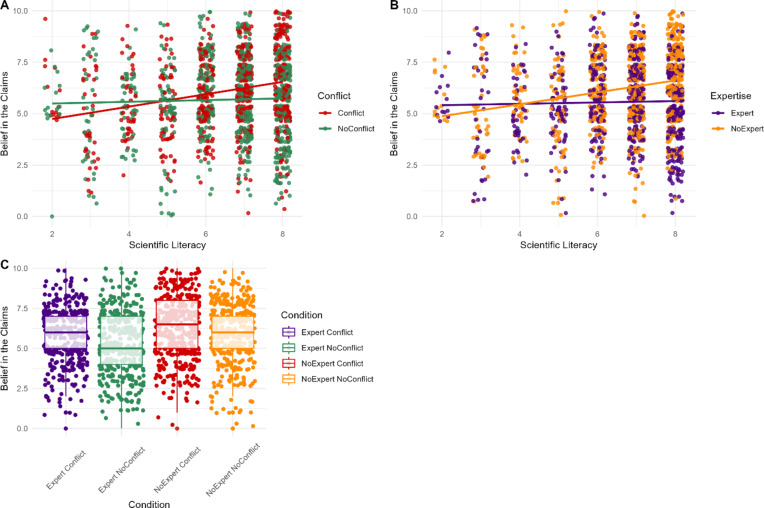
Participants’ ratings of belief in the science claims based on their scientific literacy scores and the presence of a vested interest (**A**) or expertise (**B**) and by conflict and expertise (**C**) for the *disagreeing* individual. *Note*. For panel (**A**) red indicates participant responses when the individual disagreeing with the scientist’s claim did have a conflict of interest, while green indicates participant responses when they did not have a conflict of interest. Similarly, for panel (**B**) orange points represent participant responses when the individual disagreeing with the scientist’s claim lacked relevant expertise, whereas purple points represent participant responses when they possessed relevant expertise.

Consistent with H3B and H4B, the interactions between expertise and scientific literacy (*β* = 0.23, *SE* = 0.07, *p* < .001), and conflict and scientific literacy (*β* = -0.31, *SE* = 0.07, *p* < .001), were significant. The effect of the *disagreeing* individuals’ vested interest or domain-relevant expertise were moderated by scientific literacy such that only those with greater scientific literacy were sensitive to the presence of conflicting interests or domain-specific expertise (Fig. 3). Scientific literacy also predicted belief scores independent of conflict and expertise (*β* = 0.18, *SE* = 0.09, *p* = .020). The interaction between conflict and expertise was non-significant when predicting belief (RQ2; *p* = .828).

## Experiment two

### Methods

After Study 1, we recruited a second, online sample via Prolific Academic to test replicability in a general population without reference to specific country or media systems. Participants had to be fluent in English and have a 100% approval rating. To reflect the initial sample, we recruited 110 participants, with participants reimbursed 2.55 pounds consistent with Prolific Academic rates of pay.

Our sample consisted of 110 participants (*n* = 46 male; *n* = 56 female; *n* = 1 non-binary; *n* = 1 prefer not to say) with a mean age of 30 years (*SD* = 9.16). All participants had a high school education, and the majority had completed an undergraduate degree (*n* = 48), up to a PhD (*n* = 2). Across the sample, 22 nationalities were represented from countries spanning Europe, Africa, North and South America, Australasia, Asia, and the Middle East. The largest participant groups came from South Africa (*n* = 44). A full breakdown by education and nationality is available on the OSF.

The methods and data analysis for the study were otherwise identical to Experiment 1, as were the predictions and research question.

## Results

Multiple-choice scores suggested moderate retention of article details (*M* = 12.33; *SD* = 1.97).

### Trustworthiness of claiming scientist

H1A was not supported, as the effect of conflict on trust was non-significant (*β* = -0.13, *SE* = 0.43, *p* = .673), suggest participants were not sensitive to vested interests when assessing the trustworthiness of the *claiming* scientist.

H2A was supported, with a significant effect of expertise on trust scores (*β* = -0.05, *SE* = 0.41, *d* = 0.26, *p* < .001). Trustworthiness of the *claiming* scientist was rated significantly higher when the *disagreeing* individual had non-domain-specific expertise (*M* = 6.55, *SD* = 2.06), than domain-specific expertise (*M* = 6.03, *SD* = 1.92).

**Fig. 4:**
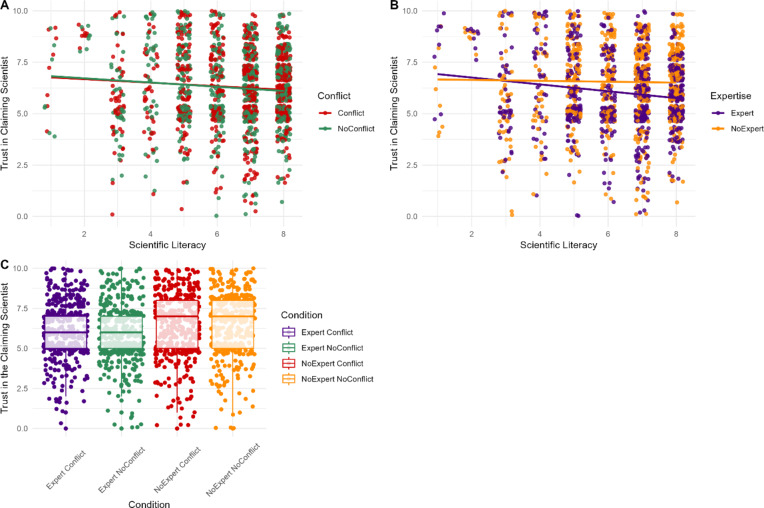
Participants’ ratings of trust in the reporting scientist based on their level of scientific literacy and the presence of a vested interest (**A**) or expertise and vested interest (**B**) and by conflict and expertise (**C**) for the *disagreeing* individual. *Note*. For panel (**A**) the red points indicate individual participants mean trust scores when the individual disagreeing with the scientist’s claim had a conflict of interest, whereas the green points depict their mean trust scores when they did not have a conflict of interest. Similarly, for panel (**B**) orange points represent participant responses when the individual disagreeing with the scientist’s claim lacked relevant expertise, whereas purple points represent participant responses when they possessed relevant expertise.

H1B was not supported, as the interaction between conflict and scientific literacy was not significant (*p* = .998). However, H2B was supported, with a significant interaction between expertise and scientific literacy (*β* = 0.13, *SE* = 0.07, *p* = .001), such that only participants with greater scientific literacy were sensitive to expertise when assessing trustworthiness in the *claiming* scientist. The interaction between conflict and expertise was also significant when predicting trust (RQ1; *β* = 0.19, *SE* = 0.61, *p* = .006; Fig. 4).

### Belief in the claim

H3A was not supported, as conflict had no statistically significant effect on belief (*p* = .224).

H4A was supported, with a significant small effect of expertise (*β* = -0.55, *SE* = 0.52, *d* = 0.14, *p* < .001). When the *disagreeing* individual possessed domain-specific expertise, participants’ belief ratings were lower (*M* = 5.94, *SD* = 2.14), than when they lacked domain-specific expertise (*M* = 6.24, *SD* = 2.14).

We found no support for H3B, H4B, or RQ2, as the interactions between expertise and scientific literacy (*p* = .075), conflict and scientific literacy (*p* = .728), and expertise and conflict (*p* = .069), were non-significant.

**Fig. 5:**
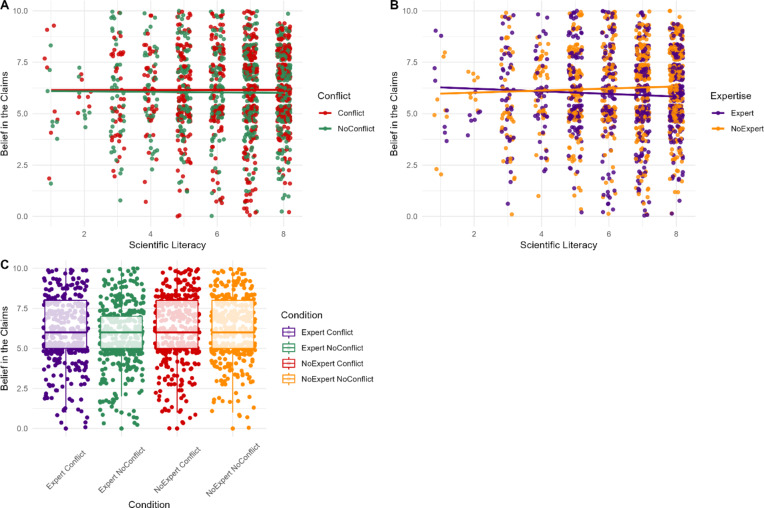
Participants’ ratings of belief in the science claims based on their scientific literacy scores and the presence of a vested interest (**A**) or expertise (**B**) and by conflict and expertise (**C**) for the *disagreeing* individual. *Note*. For panel (**A**) red indicates participant responses when the individual disagreeing with the scientist’s claim did have a conflict of interest, while green indicates participant responses when they did not have a conflict of interest. Similarly, for panel (**B**) orange points represent participant responses when the individual disagreeing with the scientist’s claim lacked relevant expertise, whereas purple points represent participant responses when they possessed relevant expertise.

## Experiment three

### Methods

Given the differences in results across experiments one and two, we ran a third experiment with an online Australian sample to be more comparable to our initial Australian student sample, and to test whether expertise and conflict also impacted trust in the *disagreeing* individual.

### Sample

We recruited a third, Australian sample via Prolific Academic to test replicability in a general population without reference to specific country or media systems. Participants had to be fluent in English and have a 100% approval rating. To reflect the initial sample, we recruited 105 participants, with participants reimbursed 2.55 pounds consistent with Prolific Academic rates of pay.

Our sample consisted of 105 participants (*n* = 53 male; *n* = 51 female; *n* = 1 non-binary) with a mean age of 37.2 years (*SD* = 10.4). All participants had a high school education, and the majority had completed an undergraduate degree (*n* = 38), up to a PhD (*n* = 4).

### Measures

In addition to the measures in Experiment 1 and Experiment 2, we also measured trust in the disagreeing scientist, scientific reasoning, and conducted two manipulation checks per claim on expertise and conflict of interest.

Trust in the disagreeing scientist was measured using a single item, e.g., “How trustworthy is Professor Y (Trust in disagreeing individual)?” From 0 = *Not at all trustworthy* through to 10 = *Completely trustworthy.*

Scientific reasoning was measured using the Scientific Reasoning Scale^42^. Participants assessed the truth or falsity of scientific claims in response to 11 brief scientific scenarios, such as “A researcher develops a new method for measuring the surface tension of liquids. This method is more consistent than the old method. True or False? The new method must also be more accurate than the old method.” Scores were summed to create a score for each participant between 0 and 11.

For the manipulation checks, participants were asked “Does Professor Y (disagreeing individual) have the appropriate expertise to comment on Professor X’s research?” (Expertise) and “Does Professor Y (disagreeing individual) have a conflict of interest (i.e., vested interest) in Professor X’s research that may affect their disagreement?” (Conflict). Participants responded Yes or No to each.

### Design and analysis

For Experiment 3 we randomised the presentation of news articles for each participant. As in Experiments 1 and 2, analysis was conducted using linear mixed-effects models in R. Interactions with scientific literacy were tested using scores on the scientific reasoning scale. Given the new measures, we added the following predictions:

#### H5A

The perceived trustworthiness of a *disagreeing* individual will be lower when the *disagreeing* individual has a conflicting interest, compared with when they have no conflicting interest.

#### H5B

There will be a significant interaction between scientific literacy and conflict, such that, as people’s scientific literacy increases, conflict will have a greater impact on the perceived trustworthiness of the individuals disagreeing with the claim.

#### H6A

The perceived trustworthiness of a *disagreeing* individual will be higher when the *disagreeing* individual is a domain expert, compared with when they are not a domain expert.

#### H6B

There will be a significant interaction between scientific literacy and expertise, such that, as people’s scientific literacy increases, the expertise of the individual disagreeing with the claim will have a greater impact on the perceived trustworthiness of the individual disagreeing with the claim.

#### RQ3

Is there an interaction between expertise and conflict, such that when the individual disagreeing with a claim lacks expertise and has a conflict this will impact trust in the disagreeing individual more than either factor in isolation?

## Results

Manipulation check scores indicated participants were more aware of the *disagreeing* individual’s expertise (*M* = 13.87, *SD* = 2.65) than conflict (*M* = 11.96, *SD* = 2.92).

### Trustworthiness of claiming scientist

H1A was not supported, as the effect of conflict was non-significant (*p* = .211), suggest participants were not sensitive to vested interests when assessing trust in the *claiming* scientist.

H2A was supported, with a significant small effect of expertise on trust (*β* = -0.02, *SE* = 0.28, *d* = 0.27, *p* < .001). Trust in the *claiming* scientist was higher when the *disagreeing* individual had unrelated expertise (*M* = 7.05, *SD* = 1.89), than domain-specific expertise (*M* = 6.55, *SD* = 1.78).

**Fig. 6:**
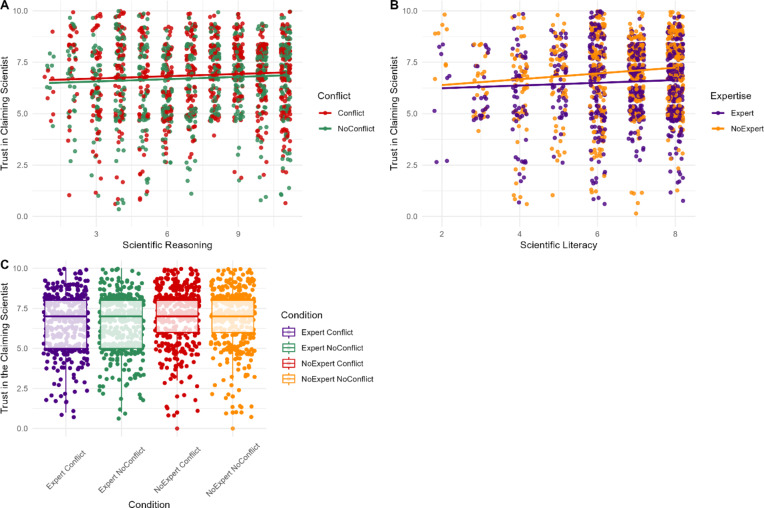
Participants’ ratings of trust in the *claiming* scientist based on their level of scientific reasoning and the presence of a vested interest (**A**) or expertise and vested interest (**B**) and by conflict and expertise (**C**) for the *disagreeing* individual. *Note*. For panel (**A**) red indicates participant responses when the individual disagreeing with the scientist’s claim did have a conflict of interest, while green indicates participant responses when they did not have a conflict of interest. Similarly, for panel (**B**) orange points represent participant responses when the individual disagreeing with the scientist’s claim lacked relevant expertise, whereas purple points represent participant responses when they possessed relevant expertise.

H1B was not supported, with a non-significant interaction between conflict and scientific reasoning (*p* = .428). However, H2B was supported, with a significant interaction between expertise and scientific reasoning (*β* = 0.07, *SE* = 0.03, *p* = .019). Only participants with greater scientific reasoning were sensitive to expertise when assessing trust in the *claiming* scientist (Fig. 6). The interaction between conflict and expertise was non-significant (RQ1; *p* = .851).

### Belief in the claim

H3A was not supported, as conflict’s effect on belief was non-significant (*p* = .633).

H4A was supported, with a significant small effect of expertise on belief (*β* = -0.29, *SE* = 0.37, *d* = 0.24, *p* = .007). When the *disagreeing* individual possessed domain-specific expertise, participants’ belief ratings were lower (*M* = 6.37, *SD* = 1.91), than unrelated expertise (*M* = 6.83, *SD* = 1.97).

**Fig. 7:**
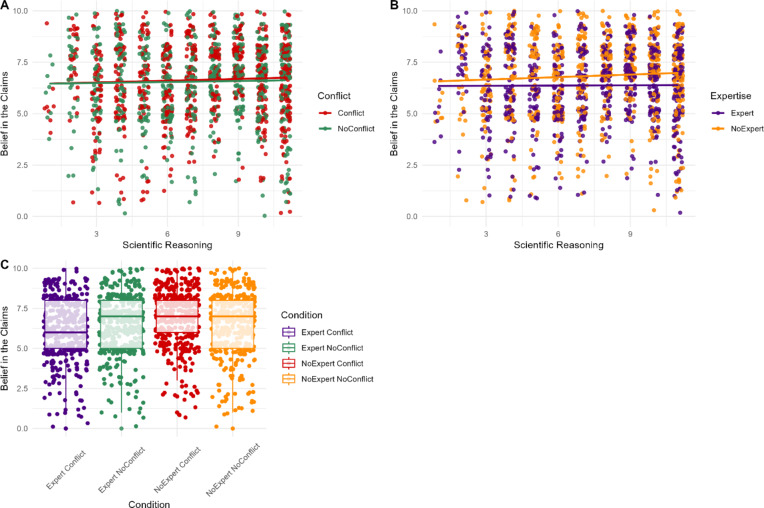
Participants’ ratings of belief in the science claims based on their scientific reasoning scores and the presence of expertise (**A**) or expertise and conflict (**B**) and by conflict and expertise (**C**) for the *disagreeing* individual. *Note*. For panel (**A**) red indicates participant responses when the individual disagreeing with the scientist’s claim did have a conflict of interest, while green indicates participant responses when they did not have a conflict of interest. Similarly, for panel (**B**) orange points represent participant responses when the individual disagreeing with the scientist’s claim lacked relevant expertise, whereas purple points represent participant responses when they possessed relevant expertise.

We found no support H3B and H4B, as the interactions between expertise (*p* = .175) and conflict (*p* = .652), with scientific reasoning were non-significant. The interaction between conflict and expertise was significant (RQ2: *β* = -0.20, *SE* = 0.05, *p* = .039). Where the *disagreeing* individual had domain-specific expertise and vested interest, participants reported lower belief scores (Fig. 7).

### Trustworthiness of disagreeing scientist

H5A was supported, as conflict’s effect on trust in the *disagreeing* individual was significant (*β* = 0.67, *SE* = 0.56, *d* = 0.40, *p* = .002). Participants reported less trust in the *disagreeing* individual when they had a conflict (*M* = 3.96, *SD* = 2.50), than when they did not (*M* = 4.95, *SD* = 2.49).

H6A was supported, with a significant large effect of expertise on trust (*β* = -1.18, *SE* = 0.56, *d* = 0.93, *p* < .001), such that the *disagreeing* individual was rated less trustworthy when they had unrelated expertise (*M* = 3.39, *SD* = 2.41), than expertise relevant to the claim (*M* = 5.52, *SD* = 2.19).

**Fig. 8:**
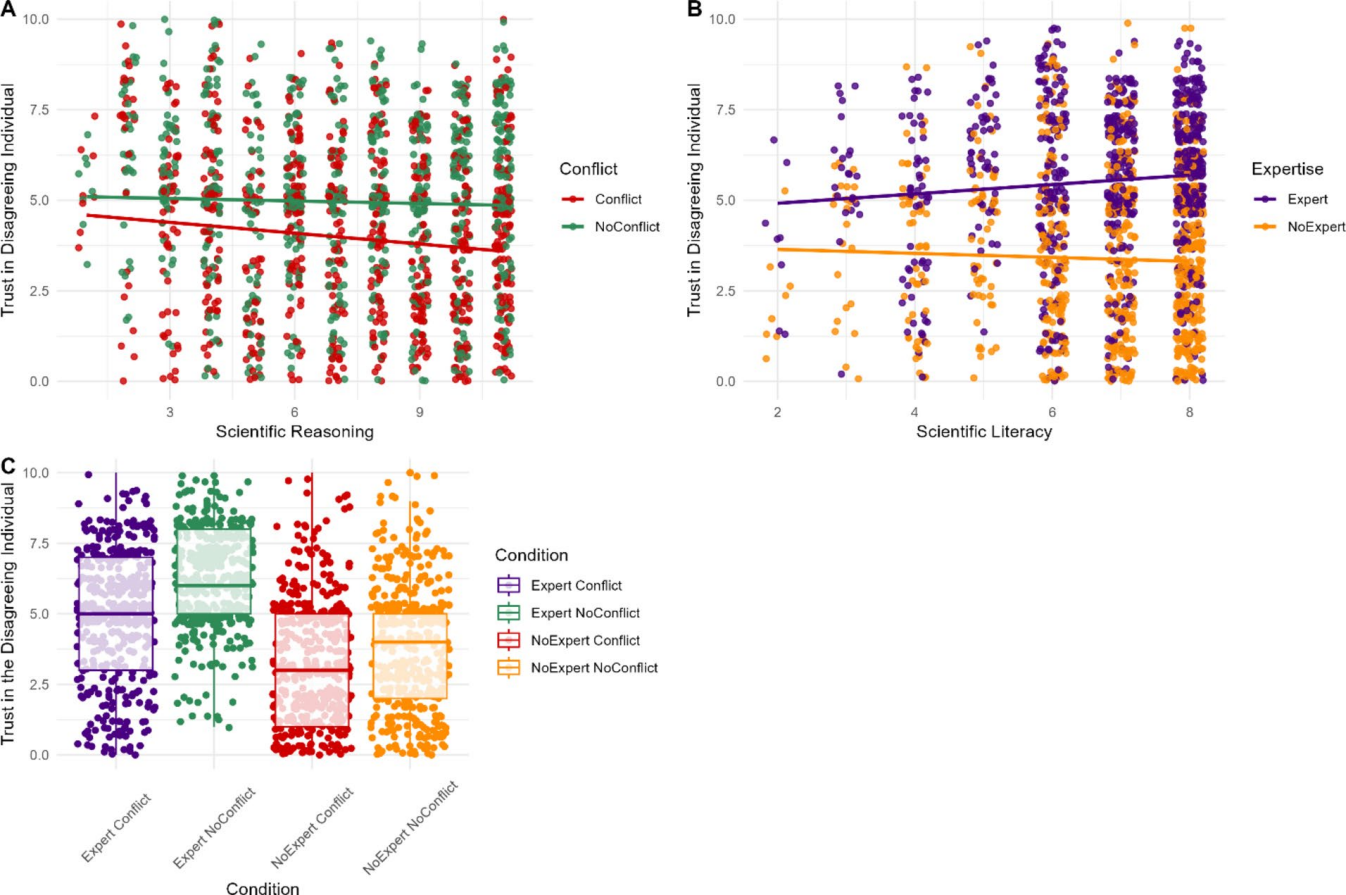
Participants’ ratings of trust in the *disagreeing* scientist based on their level of scientific reasoning and the presence of a vested interest (**A**) or expertise (**B**) and by conflict and expertise (**C**) for the *disagreeing* individual. *Note*. For panel (**A**) red indicates participant responses when the individual disagreeing with the scientist’s claim did have a conflict of interest, while green indicates participant responses when they did not have a conflict of interest. Similarly, for panel (**B**) orange points represent participant responses when the individual disagreeing with the scientist’s claim lacked relevant expertise, whereas purple points represent participant responses when they possessed relevant expertise.

H5B and H6B were supported, with significant interactions between conflict and scientific reasoning (*β* = 0.11, *SE* = 0.04, *p* = .015), and expertise and scientific reasoning (*β* = -0.07, *SE* = 0.04, *p* = .019). Participants with greater scientific reasoning were more sensitive to conflict or expertise (Fig. 8). There was no interaction between conflict and expertise on trust in the *disagreeing* scientist (*p* = .160).

## Discussion

Across three 2 × 2 factorial experiments, we investigated how *disagreeing* individuals’ characteristics influence trust in *claiming* scientists and belief in their claims, and whether scientific literacy moderated these effects. Participants read short news articles featuring a scientific claim and a disagreeing source whose traits were manipulated using Mayer et al.’s ABI model^24^, specifically expertise (ability) and vested interests/conflict (integrity). As summarised in Table 1, our findings demonstrate that *disagreeing* individuals can sway evaluations of *claiming* scientists’ trustworthiness and claims’ credibility. However, while participants were robustly sensitive to domain-specific expertise, only those in the student sample with high scientific literacy were sensitive to conflicts-of-interest, while general samples remained largely insensitive to cues indicating vested interests. Our third study suggests participants were sensitive to vested interests, but only took them into account when assessing the *disagreeing* individuals’ trustworthiness. Although scientific literacy consistently moderated expertise’s impact on trust in the *claiming* individual, and *disagreeing* individual in Study 3, as a moderator of expertise’s impact of belief in science claims, or of conflict’s influence, it was less consistent.

**Table 1:** Summary of hypotheses/research questions by sample. H5A onwards was only relevant for the Australian sample (Study 3).

Prediction	Student	Online	Australian
**H1A**: Conflict predicting *Claiming* Scientist Trustworthiness	X	ns	ns
**H1B**: Conflict*Scientific Literacy predicting *Claiming* Scientist Trustworthiness	X	ns	ns
**H2A**: Expertise predicting *Claiming* Scientist Trustworthiness	X	X	X
**H2B**: Expertise*Scientific Literacy predicting *Claiming* Scientist Trustworthiness	X	X	X
**RQ1**: Conflict*Expertise predicting *Claiming* Scientist Trustworthiness	ns	X	ns
**H3A**: Conflict predicting Belief	X	ns	ns
**H3B**: Conflict and Scientific Literacy predicting Belief	X	ns	ns
**H4A**: Expertise predicting Belief	X	X	X
**H4B**: Expertise and Scientific Literacy predicting Belief	X	ns	ns
**RQ2**: Conflict and Expertise predicting Belief	ns	ns	X
**H5A**: Conflict predicting Trust in *Disagreeing* Individual			X
**H5B**: Conflict*Scientific Literacy predicting *Disagreeing* Individual Trustworthiness			X
**H6A**: Expertise predicting *Disagreeing* Individual Trustworthiness			X
**H6B**: Expertise*Scientific Literacy predicting *Disagreeing* Individual Trustworthiness			X
**RQ3**: Expertise*Conflict predicting *Disagreeing* Individual Trustworthiness			ns

Our samples provide partial support to our hypotheses, but indicate insensitivity toward vested interests when evaluating scientific disagreements, and a contingent role of scientific literacy. Across all samples, the *disagreeing* individuals’ domain-specific expertise impacted judgements of trust and belief in the *claiming* expert and their claim. The interaction between expertise and scientific literacy when predicting trust in the *claiming* scientist was also present across all samples, indicating a robust effect of expertise, and of scientific literacy in enhancing awareness of *disagreeing* individuals’ domain-specific expertise when assessing *claiming* expert’s trustworthiness.

However, only the student sample were sensitive to vested interest when evaluating belief, and only students high in scientific literacy were sensitive to conflicts when evaluating *claiming* scientists’ trustworthiness. This suggests a baseline level of trustworthiness when a *disagreeing* individual has no conflict and scientific experts are disagreed with by other subject-matter experts. Both online samples were insensitive to conflicting interests when assessing *claiming* scientists’ trustworthiness or belief in their claims, irrespective of scientific literacy. Previous research manipulating the *claiming* individuals’ traits has found variable effects of conflict^13–15^, suggesting this insensitivity may be an issue irrespective of which side an expert holds in a disagreement (i.e., *claiming* or *disagreeing*). However, our third study suggests conflicts may impact judgements of trust in the individual with the conflict (here, the disagreeing individual), without impacting judgements of their claims or interlocutors. Scientific reasoning also moderated both expertise and conflict, increasing their effects when assessing belief in the *disagreeing* individual in Study 3. Together, our results therefore suggest scientific literacy is influential in evaluating scientific disagreements, with those higher in scientific literacy sometimes more cognisant of cues to the credibility of opposing views, particularly expertise. However, this influence is variable, particularly when considering vested interests. While we were interested in the traits in isolation, inconsistent moderation effects between expertise and conflict were found across the samples, some of which were unexpected (e.g., an expert with a vested interest lowering belief in the claim relative to other conditions, Study 2). These effects may merit further investigation.

Our findings suggest three key takeaways. Firstly, disagreement from expert sources can negatively impact people’s trust in scientists and belief in science claims. Secondly, vested interests of a *disagreeing* individual exert little to no influence over evaluations of scientific disagreement. Even among those sensitive to conflict (i.e., highly scientifically literate participants in our student and Australian sample), expertise exerted a greater influence than conflict on judgements of trust in *claiming* scientists and belief in their claims. Additionally, our manipulation check in Study 3 suggests participants were less sensitive to vested interests than to expertise. Third, scientific literacy can enhance awareness to expertise and conflict cues, but is not a panacea, and likewise exerts greater influence in attending expertise than conflict. Education in scientific research methods, potentially higher among our psychology student sample, may increase sensitivity to conflict’s impact on science claims and credibility, though needing further research. Overall, these results indicate consequences for science news consumption in modern media environments where disagreement is amplified, expertise can be difficult to verify, and individuals often speak from positions of vested interest. Critically, our findings indicate more public understanding is needed around conflict in scientific disagreement.

Our work builds on past research^13–16^ by investigating traits of the *disagreeing* individual and their interaction with scientific literacy. By manipulating the *disagreeing* individuals’ characteristics while measuring trust in the *claiming* scientist and belief in their claim, our findings demonstrate one interlocutor’s traits can influence perceptions of the other. Study 3 suggests some variance in this influence though, with expertise being more influential on assessments of others than conflict, which exerts greater influence over assessments of the vested individual. Future studies could explore how different combinations of claimant and conflicting source characteristics influence trust and belief in both parties and claims. We also build on previous correlational findings^35,36^ regarding scientific literacy. While variable, our results suggest future research manipulating *claiming* or *disagreeing* individuals’ characteristics may benefit from including individual differences such as scientific literacy, to better understand how these traits influence assessments of scientific disagreement.

Trust and belief in scientific claims influence people’s behavioural choices, including compliance with evidence-based policy^11,23^. Our findings therefore indicate a need to improve public understanding and evaluation of scientific disagreements. This improvement could include interventions at the level of both journalism and communication professionals, and individuals. Individual level intervention could include training that improves scientific literacy and awareness of cues to expertise and vested interests. Given the variability and frequent insensitivity to vested interests across our samples, training should particularly emphasise how conflict can impact the credibility of scientific claims and disagreements. Many people access scientific claims via news and other mass or social media, and journalistic reporting can sway trust in science^3,4^ yet often tries to balance scientific claims with differing opinions and sources, without considering or reporting subject-matter expertise and/or conflict^43^. Accordingly, implementing reporting standards could ensure that cues to expertise and integrity are readily apparent for both *claiming* and *disagreeing* individuals, and potentially ‘nudge’ audiences to considering these cues when evaluating belief and trustworthiness. However, given those lower in scientific literacy may be less sensitive to or not attend cues to expertise; and that audiences may not be sensitive to conflicts irrespective of scientific literacy, journalists and communicators should consider the validity of their sources before reporting disagreements, and not see disclosure of expertise and/or vested interests as a sufficient measure of responsible reporting.

### Strengths and limitations

We deliberately selected non-partisan topics, as most scientific claims people are exposed to are not politically charged^40^, and we wanted to investigate the impact of expertise, conflict, and scientific literacy on judgements of trust and belief in scientific disagreements without confounding by political motives, e.g., identity motivated responding^41^. However, this design choice may limit generalisability to political claims. Future research could explore impacts on polarised topics, particularly as scientific literacy may enhance identity-motivated reasoning.

While our scientific literacy measure has been used in research on scientific claims and misinformation^41^, it concerns general scientific knowledge and is subject to ceiling effects, including within our student sample^44^. We therefore used the more robust scientific reasoning scale^42^ in Study 3. Our results may also be influenced by differences across the samples, particularly by the student sample. First-year psychology undergraduates are more likely to have received research methods training, often including evaluation of scientific research, which may explain the greater influence of conflict in Study 1. Additionally, all three samples may differ from the general population, the first being Australian psychology students, the latter two being self-selected internet users on Prolific, with Study 2 having a large portion of South African participants. Nevertheless, that expertise influenced judgements of trust and belief across all three studies suggests a relatively robust effect.

Finally, responses to our multiple-choice and manipulation check questions suggest participants were attentive to article details but did not have perfect recall. However, this pattern of responding likely reflects reading in a naturalistic setting and indicates the importance of media and scientific literacy training to increase awareness of credibility cues in scientific news. While our manipulations were sometimes subtle, which may have reduced the size of effects, this is often reflective of how conflicts and expertise are present in public scientific disagreements. That participants did not always register vested interests, or non-domain-specific expertise, highlights the need for greater public awareness of these cues.

## Conclusions

Understanding how science claims are received amidst disagreement is vital for effective science communication. Previous research has predominantly considered how scientific consensus messaging^12^ or claimant characteristics impact trust^39^. We extend these findings by demonstrating how *disagreeing* sources’ traits impact trust in scientists and belief in their claims, and the importance of individual differences like scientific literacy.

Our findings indicate a *disagreeing* source can substantially influence people’s trust in scientists and belief in scientific claims. However, participants appeared relatively insensitive to cues indicating conflicting interests. While scientific literacy moderated expertise’s impact on trust and belief across all samples, it only moderated conflict’s impact in the student sample. However, our third study, the only one measuring trust in the *disagreeing* scientist found conflict impacted trust, and this effect was moderated by scientific literacy. These findings indicate the importance of including individual differences, like scientific literacy, when investigating evaluations of scientific disagreement and communication.

Collectively, our results highlight the need for communicators—particularly journalists—to make credibility cues, including expertise and vested interests, more transparent when reporting scientific disagreements. Scientific literacy can foster greater sensitivity to these cues, but does not guarantee audiences will notice or interpret them. Educational and training initiatives aimed at strengthening scientific literacy, alongside enhanced journalistic practices, may therefore bolster public understanding of—and trust in—science. By helping people to recognise and interpret disagreements in media coverage, such efforts could ultimately promote more informed decision-making in science-related domains.

## Data Availability

The datasets generated and/or analysed during the current study are available in the Open Science Framework repository: https://osf.io/9kbe4/.
